# Investigating methylparaben’s oxidative stress effects on rainbow trout blood, liver, and kidney toxicity

**DOI:** 10.4102/ojvr.v92i1.2200

**Published:** 2025-03-07

**Authors:** Mert Calisir, Gokhan Nur, Emrah Caylak

**Affiliations:** 1 Department of Chemical, Biological, Radiological and Nuclear Threats Management, Faculty of Engineering and Natural Sciences, Iskenderun Technical University, Hatay, Türkiye; 2 Department of Biomedical Engineering, Faculty of Engineering and Natural Sciences, Iskenderun Technical University, Hatay, Türkiye; 3 Department of Biochemistry, Faculty of Medicine, Girne American University, Girne, Cyprus

**Keywords:** *Oncorhynchus mykiss*, methylparaben, hepatotoxicity, nephrotoxicity, GSH-Px, MDA, urea and uric acid

## Abstract

The widespread use of parabens has led to their accumulation in aquatic environments. This study examined the effects of methylparaben on rainbow trout, dividing 96 fish into control and treatment groups (1 mg/L, 5 mg/L, and 8 mg/L). Results showed dose-dependent weight loss, altered hepatosomatic indices, increased serum urea, uric acid, and Malondialdehyde (MDA) levels, and decreased Glutathione Peroxidase (GSH-Px) activity. Histopathological analysis revealed liver and kidney abnormalities in treated groups, including hepatocyte degeneration, proliferation in the bile duct, glomerular atrophy, reduced haematopoietic tissue, increased melanomacrophage centres, necrosis and fibrosis.

**Contribution:**

These findings highlight methylparaben’s toxic effects, emphasising the need for stricter regulations and further research to safeguard aquatic ecosystems and understand its impact on aquatic organisms.

## Introduction

Since the 20th century, rapid growth in the cosmetics and food industries has driven the demand for parabens, widely used as biocides in food, pharmaceuticals, and personal care products for their preservative properties. Parabens are esters of p-hydroxybenzoic acid (p-HBA) that differ from each other according to the type of substituent, which can be an alkyl chain or an aromatic ring. Parabens are classified as endocrine-disrupting chemicals that can interfere with normal thyroid functioning, affecting the proper regulation of the biosynthesis of thyroid hormones controlled by the hypothalamic-pituitary-thyroid axis. Despite their benefits, parabens such as methylparaben and propylparaben raise environmental concerns due to their endocrine-disrupting effects, impacting thyroid function and causing oestrogenic reactions in aquatic organisms, even at low concentrations. The increasing presence of parabens in water systems highlights the need for stricter regulation and research. This could lead to alarming levels of parabens in different ecosystems and cause complications for human and animal health (Atli 2021; Azeredo et al. 2023; Lincho, Martins & Gomes 2021; Pirinc & Turkoglu 2016). There are many studies on parabens in fish. Although the number of studies with methyl paraben is higher than the others, other fish have been studied more than trout. There are also a few studies where rainbow trout and methyl paraben were studied together (Dasmahapatra, Chatterjee & Tchounwou 2024). In vitro methods such as hepatocyte culture were used in these studies. Our study is unique in terms of showing the histological and biochemical changes caused by methyl paraben in trout, which is a bioindicator in showing water pollution. This study investigates methylparaben’s oxidative stress-mediated haemotoxicity, hepatotoxicity, and nephrotoxicity in rainbow trout (*Oncorhynchus mykiss*).

## Research methods and design

### Experimental design

Fish (152.25 ± 25.10 g, 20.09 ± 1.11 cm) were acclimated for 1 week in laboratory aquaria under controlled conditions (12 °C – 19 °C, oxygen ≥ 7 mg/L, pH 6.5–7.5). They were fed dry pellets and fasted for 24 h before the experiments. Four groups were tested: a control group and three groups exposed to 1 mg/L, 5 mg/L, and 8 mg/L methylparaben (Barse et al. 2010; Dasmahapatra et al. 2024; De Carvalho Penha et al. 2021; Silva et al. 2018; Terasaki, Makino & Tatarazako 2009, United States [US] EPA 2008). Daily water changes and treatments maintained clean conditions. After 21 days, fish were anaesthetised (MS222, 50 mg/L) (Ross & Ross 2008), and blood samples were measured and collected. Liver and kidney tissues were sampled for histopathological analysis, allowing a systematic assessment of methylparaben’s impact on key biological systems. Fish weights were recorded on day 0 and after the study to monitor progression. Liver weights were measured at both time points, and the hepatosomatic index (HSI) was calculated using the formula:


HSI=(liver weight/body weight)×100. [Eqn 1]

### Histopathological and biochemical analysis

On day 21, a dissection of liver and kidney tissues was conducted for histopathological analysis. Samples were fixed in 10% buffered formalin for 48 h, rinsed, and processed through alcohol and xylene treatments before embedding in paraffin. Thin sections (4 µm – 5 µm) were cut and stained with haematoxylin-eosin for microscopic examination (Zeiss Axio Imager 2) (Presnell & Schreibman 1997). Tissue alterations were graded as absent (–), mild (+), moderate (++), or severe (++++) relative to the control group.

Blood samples were collected post-anaesthesia (MS222, 50 mg/L) (Ross & Ross 2008) and centrifuged to separate serum stored at –20 °C. Serum urea and uric acid were analysed using a Hitachi-Roche Diagnostics Cobas 6000 biochemical analyser. Malondialdehyde (MDA) and Glutathione Peroxidase (GSH-Px) activity were quantified with specific detection kits to assess oxidative stress and antioxidant status.

### Statistical analysis

Data were analysed using Statistical Package for Social Sciences (SPSS) 22.0. Normality was assessed, and parametric (analyses of variance [ANOVA] with Tukey honestly significant difference [HSD]) or non-parametric (Kruskal-Wallis) tests were applied as appropriate. Statistical significance was set at *p* < 0.05, with mean ± standard error results.

### Ethical considerations

Ethical clearance to conduct this study was obtained from the Iskenderun Technical University Faculty of Aquaculture Ethics Committee of Animal Experiments (No. ISTE-SUHADYEK/2024-12332).

## Results

HSI values were monitored to assess fish health (see Table 1). No significant difference was observed between groups on day 1 (*p* > 0.05), and the control group showed no significant difference from methylparaben-treated groups on day 21 (*p* > 0.05). However, a significant difference was found between the lowest (1 mg/L) and highest (8 mg/L) methylparaben doses (*p* < 0.05).

**TABLE 1:** Changes in liver somatic index caused by methylparaben in *Oncorhynchus mykiss.*

Groups	HSI (*x* ± s.e.)	
	0 Day	21 Day
Control group	1.21 ± 0.020†	1.35 ± 0.029†,‡
1 mg/L group	1.28 ± 0.028†	1.30 ± 0.026‡
5 mg/L group	1.22 ± 0.019†	1.41 ± 0.016†,‡
8 mg/L group	1.20 ± 0.012†	1.44 ± 0.030‡

*n*, number of subjects in the group, s.e., standard error; HSI, hepatosomatic index.

†,‡ , The difference between group averages with different symbols in the same column is significant (*p* < 0.05).

Over 21 days, weight gain progressively decreased with higher methylparaben doses. The control group showed the highest weight increase (59.12 g, 40.49%), followed by 1 mg/L (56.12 g, 35.74%), 5 mg/L (46.37 g, 28.31%), and 8 mg/L (39.38 g, 27.51%). Higher methylparaben concentrations significantly reduced weight gain and percentage increase, particularly in the 5 mg/L and 8 mg/L groups (Table 2).

**TABLE 2:** Weight gain (g) and percentage rate in control and methylparaben-applied in *Oncorhynchus mykiss.*

Groups	Weight gain (g)	Weight gain rate (%)
Control	59.12	40.49
1 mg/L	56.12	35.74
5 mg/L	46.37	28.31
8 mg/L	39.38	27.51

**FIGURE 1:**
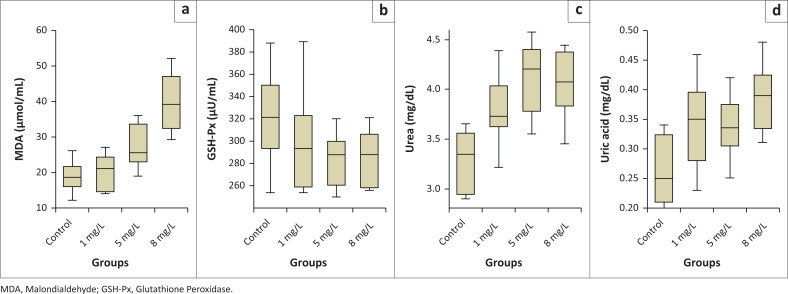
(a) MDA, (b) GSH-Px, (c) urea, and (d) uric acid levels in methyl paraben-treated in *Oncorhynchus mykiss*.

In Table 3 and Figure 1, biochemical analysis revealed that methylparaben exposure increased MDA levels (control: 18.75 µmol/mL; 1 mg/L: 20.0 µmol/mL; 5 mg/L: 27.37 µmol/mL; 8 mg/L: 39.75 µmol/mL). While no significant difference was observed between the control and 1 mg/L groups (*p* > 0.05), MDA levels significantly increased in the 5 mg/L and 8 mg/L groups compared to the control (*p* < 0.05). Glutathione Peroxidase activity decreased with higher doses (control: 321.62 µU/mL; 1 mg/L: 299.25 µU/mL; 5 mg/L: 283.12 µU/mL; 8 mg/L: 285.12 µU/mL), but this decrease was not statistically significant (*p* > 0.05).

**TABLE 3:** Biochemistry data from rainbow trout in the groups.

Groups	MDA (*x* ± s.e.)	GSH-Px (*x* ± s.e.)	Urea (*x* ± s.e.)	Uric acid (*x* ± s.e.)
Control	18.75 ± 1.55†	321.62 ± 14.83§	3.28 ± 0.11‡	0.26 ± 0.02‡
1 mg/L	20.00 ± 1.83†,‡	299.25 ± 16.72§	3.79 ± 0.12§	0.34 ± 0.02‡,§
5 mg/L	27.37 ± 2.21‡	283.12 ± 8.74§	4.11 ± 0.13§	0.33 ± 0.01‡,§
8 mg/L	39.75 ± 3.03§	285.12 ± 9.00§	4.05 ± 0.12§	0.38 ± 0.02§

s.e., standard error; MDA, Malondialdehyde; GSH-Px, Glutathione Peroxidase; *n*, number of subjects in the group.

†,‡,§ , The difference between group means with different symbols in the same column is significant (*p* < 0.05).

Serum urea levels rose significantly in treated groups compared to the control (control: 3.28 mg/dL; treated groups: 3.79 mg/dL – 4.11 mg/dL; *p* < 0.05), while differences among treated groups were insignificant. Uric acid levels increased slightly, with a significant rise only in the 8 mg/L group (control: 0.26 mg/dL; 8 mg/L: 0.38 mg/dL; *p* < 0.05).

**FIGURE 2:**
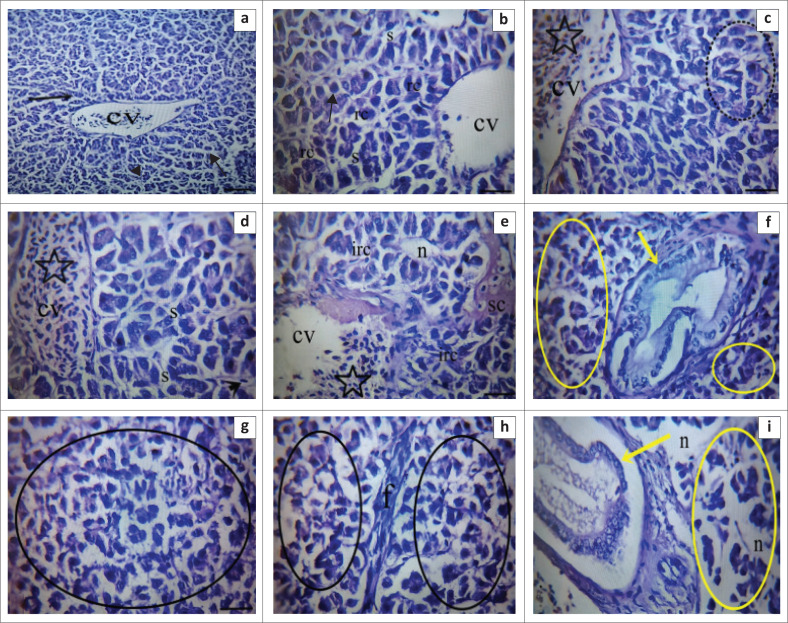
(a–i) Liver tissues of *Oncorhynchus mykiss* exposed to methylparaben doses (1 mg/L – 8 mg/L) showed dose-dependent histopathological changes, including central vein congestion, hepatocellular degeneration, sinusoidal congestion, necrosis, fibrosis, and bile duct proliferation. Control tissues appeared normal. Observed histopathological features included congestion in the central vein (cv), mild hepatocellular degeneration (black arrows), sinusoidal congestion (s), Kupffer’s star cell activation (black arrowhead), irregular remark cords (rc), necrotic areas (n), and fibrotic changes (f) in hepatocytes. Furthermore, observations encompassed degeneration and proliferation of the bile duct (yellow arrows), vacuolar and hepatocellular degeneration in the parenchymal region (yellow ring), as well as areas of necrosis and steatosis (black rings). Findings were observed at × 400 magnification using haematoxylin and eosin (H&E) staining.

Histopathological analysis of liver tissues revealed dose-dependent lesions following methylparaben exposure. Control group liver sections displayed regular hepatocyte arrangement, sinusoids, and central vein branching (Figure 2a and b). The 1 mg/L group had minimal lesions, mild hepatocyte degeneration, and occasional central vein congestion (Figure 2c and d). The 5 mg/L group showed sinusoidal congestion, necrosis, irregular hepatocyte cords, bile duct degeneration and proliferation, and vacuolar degeneration in the parenchymal region (Figure 2e and f). The 8 mg/L group exhibited severe hepatocyte degeneration, necrosis, steatosis, fibrosis, and bile duct degeneration (Figure 2g and h). Lesion severity increased with methylparaben dosage, indicating significant liver toxicity at higher exposure levels.

Examination of fish liver tissue sections indicated that higher doses of the substance increased the frequency and severity of detected lesions in the groups (Table 4).

**TABLE 4:** Methylparaben treated changes in liver tissue of *Oncorhynchus mykiss*.

Liver lesions	Control group	Methylparaben groups		
		1 mg/L	5 mg/L	8 mg/L
Hepatocellular degeneration	-	+	++	++
Degeneration-proliferation in the bile duct	-	-	+	+
Necrosis and steatosis	-	-	+	++
Irregular remark cords	+	+	++	++
Fibrosis	-	-	+	+
Sinusoidal dilatation	-	-	+	++
Congestion	-	-	++	++

*Source*: Bernet, D., Schmidt, H., Meier, W., Brkhardt-Holm, P. & Wahli, T., 1999, ‘Histopathology in fish: Proposal for a protocol to assess aquatic pollution’, *Journal of Fish Diseases* 22(1), 25–34. https://doi.org/10.1046/j.1365-2761.1999.00134.x

-, no anomaly; +, low frequency of abnormality; ++, moderate frequency of abnormality; ++++, high frequency of abnormality.

**FIGURE 3:**
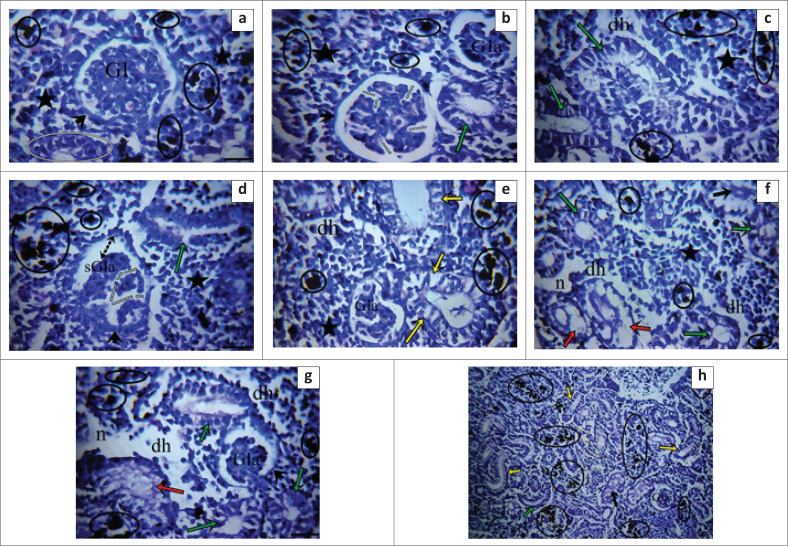
(a–h) Kidney sections from *Oncorhynchus mykiss* exposed to methylparaben showed dose-dependent damage: glomerular atrophy, tubule degeneration, necrosis, and fluid accumulation. Control tissues appeared normal, while higher doses (5 mg/L – 8 mg/L) exhibited severe glomerular atrophy, Bowman cavity enlargement, and haematopoietic tissue reduction. Glomerulus (Gl), Bowman’s capsule (arrowhead), haematopoietic tissue (asterisk), melanomacrophage centres (black ring), tubule epithelium, and lumen part (white ring), glomerular lobulation (dashed lines), glomerular atrophy (Gla), degenerations in tubule epithelium (green arrows), decrease in haematopoietic tissue (dh), severe glomerular atrophy (sGla), enlargement of Bowman’s cavity (double-sided arrow with dash), hydropic degeneration of tubule epithelium (yellow arrows), renal tubular necrosis (red arrows), necrosis (*n*), accumulation of fluid in the lumen of the tubule (black arrows). Observations were made under ×400 magnification using haematoxylin and eosin (H&E) staining.

Kidney tissue analysis revealed normal renal corpuscles and tubules in the control group, with smooth lumens and melanomacrophage centres in renal haematopoietic tissue (Figure 3). In the 1 mg/L methylparaben group, tubule epithelial degeneration and glomerular lobulation were observed. At 5 mg/L, glomerular atrophy, necrosis, hydropic degenerations, and reduced haematopoietic tissue were noted. The 8 mg/L group showed severe glomerular atrophy, tubular necrosis, Bowman cavity enlargement, fluid accumulation in tubules, and increased melanomacrophage centres, highlighting dose-dependent renal damage.

We found that the frequency and severity of lesions observed in the renal tissue were less frequent at the 1 mg/L dose but more intense at the 5 mg/L and 8 mg/L doses (Table 5).

**TABLE 5:** Tissue changes ratings of histopathological lesions in renal tissue in *Oncorhynchus mykiss.*

Kidney tissue lesions	Control group	Methylparaben groups		
		1 mg/L	5 mg/L	8 mg/L
Hydropic degeneration of the tubule epithelium	-	+	++	++
Melanomacrophage centres	+	++	++	+++
Renal tubular necrosis	-	+	++	+++
Glomerular atrophy	-	+	++	+++
Glomerular lobulation	-	-	+	++
Haematopoietic tissue reduction	-	+	+	+
Accumulation of fluid in the tubules	-	+	+	+

*Source:* Bernet, D., Schmidt, H., Meier, W., Brkhardt-Holm, P. & Wahli, T., 1999, ‘Histopathology in fish: Proposal for a protocol to assess aquatic pollution’, *Journal of Fish Diseases* 22(1), 25–34. https://doi.org/10.1046/j.1365-2761.1999.00134.x

-, no anomaly; +, low frequency of abnormality; ++, moderate frequency of abnormality; ++++, high frequency of abnormality.

## Discussion

All living beings in an ecosystem are interconnected. Water pollution affects aquatic organisms, impacting the species that rely on them for food, including humans. Evaluating the effects of chemicals from domestic, industrial, and agricultural activities on human health and aquatic life is crucial. Many everyday products contain parabens, which are preservatives but may have harmful effects. Studies show that parabens can harm the human endocrine system and aquatic ecosystems. Common parabens include methylparaben, ethylparaben, propylparaben and heptylparaben. They are widely found in consumer products and detected in various water sources, such as sewage and agricultural water, indicating their global presence. While our bodies quickly excrete parabens, they persist in aquatic environments, leading to ongoing exposure (Azeredo et al. 2023; Bernet et al. 1999; Pereira, Simões & Gomes 2023; Presnell & Schreibman 1997; Ross & Ross 2008; Yamamoto et al. 2011). Rodents and trouts, the most sensitive aquatic organisms, are used in the studies as bioindicator organisms. The toxic character of various chemical substances is examined in the biochemical, histopathological, and molecular aspects of these organisms (Deveci et al. 2015; Dogan, Deveci & Nur 2021; Nur & Deveci 2018). Paraben exposure can cause behavioural changes, nervous system disorders, hepatotoxicity, and nephrotoxicity in fish (Dasmahapatra et al. 2024).

Bedoux et al. (2012) assessed blood electrolyte values – sodium, potassium, chloride, and nitrogenous waste (urea, uric acid, blood urea nitrogen [BUN]) – before and after transplantation. Results showed stable serum sodium levels before and 12 h post-transplant but a rise immediately after. Transplanted fish had significantly higher urea, uric acid, and BUN levels than non-transplanted fish, while potassium and chloride remained unchanged. Another study found no significant differences in aspartate aminotransferase (AST), alanine aminotransferase (ALT), calcium, urea, and total protein levels across various water types in rainbow trout. However, these levels increased in summer, likely due to seasonal stress (Kelestemur & Ozdemir 2010). Additionally, urea levels among rainbow trout in different environments showed no significant differences, but uric acid levels were higher in natural habitats, possibly due to stress (Coskun, Aydin & Duman 2016).

The liver in fish is vital for metabolic processes and monitoring the effects of aquatic pollutants. It converts highly toxic ammonia (NH_3_) into urea for excretion by the kidneys. A study found that administering methylparaben and propylparaben led to increased serum AST and ALT levels and a slight, statistically insignificant decrease in serum urea levels. Histological analysis of liver tissues showed degeneration, pyknotic cells, sinusoidal enlargement and necrosis. There was evidence of tubular degeneration, hyperplasia, mononuclear cell infiltration and glomerular atrophy in kidney tissues (Inkaya & Barlas 2022). In a study conducted with male mosquitofish, severe damage was described in the liver as histopathological changes, including hepatic sinus dilatation, cytoplasmic vacuolation, cytolysis and nuclear aggregation (Ma et al. 2023).

Hu et al. (2022) examined the effects of higher doses of methylparaben on adult zebrafish over 28 days, leading to hepatocellular vacuolisation and severe liver cell membrane rupture in females. Methylparaben disrupted oxidative stress balance and significantly dysregulated lipid metabolism in the gut, blood, and liver, affecting lipid nuclear receptor transcriptions and key metabolite concentrations. Jeong et al. (2019) found the highest methylparaben levels in dolphins were in the kidney (130 ng/g), liver (120 ng/g) and stomach (80 ng/g). Another study noted lower accumulation in Brazilian guitarfish muscle (0.01 ng/g) but higher in the liver (78.52 ng/g) (Martins, Costa & Bianchini 2023).

Malondialdehyde, a key indicator of lipid peroxidation, is formed during the oxidation of membrane polyunsaturated fatty acids. It can damage macromolecules such as deoxyribonucleic acid (DNA) and proteins, leading to cellular dysfunction. Silva et al. (2018) found that glutathione activity in Nile tilapia exposed to paraben mixtures initially decreased but returned to baseline by day 12. There were no significant changes in superoxide dismutase (SOD), GSH-Px, GR activity, or catalase (CAT) and MDA levels, indicating antioxidant adaptation to non-lethal paraben levels. In contrast, Li et al. (2023) observed increased MDA levels and decreased CAT and SOD activities in zebrafish larvae exposed to butylparaben. In those exposed to a mix of propylparaben and benzisothiazolinone, SOD, CAT, and GSH-Px activities rose, while male fish showed increased vitellogenin transcription, a decreased gonadosomatic index, and an increased hepatosomatic index; female fish showed no changes in these indices.

Barse et al. (2010) found a significant increase in liver weight after administering methylparaben at doses of 0.84 mg/L, 1.68 mg/L, and 4.2 mg/L. Testicular sizes decreased in proportion to the doses. The group treated with 0.84 mg/L experienced fewer histopathological lesions than the higher doses, which showed hepatocyte necrosis and increased vacuoles. Our research indicated elevated MDA levels due to lipid peroxidation from methylparaben, alongside decreased GSH-Px enzyme levels, disrupting the oxidant-antioxidant balance. Liver enzyme activities rose, leading to an increased hepatosomatic index. Huang et al. (2024) also indicated that ethyl paraben affected the liver of rohu fish, causing necrotic areas, while butylparaben caused kidney damage in zebrafish, including glomerular atrophy and chronic nephrotoxicity. Histopathology is regarded as an efficient and sensitive method of viewing the structural changes brought about chemically, as it is reflective of the resulting biochemical and physiological changes (Dogan, Nur & Deveci 2022).

The study found elevated serum urea and uric acid levels compared to the control group, along with liver tissue degeneration, sinusoidal congestion, and bile duct changes. Similar kidney tissue issues, including tubule degeneration, increased melanomacrophage centres, and decreased haematopoietic tissue, were also observed. These findings are significant as they provide further evidence of the harmful effects of parabens on aquatic life and human health. Methylparaben, a common antimicrobial preservative, poses concerns due to its endocrine-disrupting properties and can accumulate in marine organisms, ultimately exposing humans to the food chain. Therefore, opting for paraben-free personal care products is advised for health.
